# Impact of curcumin on replicative and chronological aging in the *Saccharomyces cerevisiae* yeast

**DOI:** 10.1007/s10522-019-09846-x

**Published:** 2019-10-28

**Authors:** Karolina Stępień, Dominik Wojdyła, Katarzyna Nowak, Mateusz Mołoń

**Affiliations:** Department of Biochemistry and Cell Biology, University of Rzeszow, Zelwerowicza 4, 35-601 Rzeszow, Poland

**Keywords:** Aging, Curcumin, Hypertrophy, Oxidative stress, Yeast

## Abstract

Curcumin is a biologically active compound of vegetable origin which has a hormetic effect. Pro-health and anti-aging properties of curcumin have been known for years. The main benefit of curcumin is thought to be its anti-oxidative action. Despite vast amount of data confirming age-delaying activity of curcumin in various groups of organisms, so far little has been discovered about curcumin’s impact on cell aging in the experimental model of the *Saccharomyces cerevisiae* budding yeast. We have been able to demonstrate that curcumin significantly increases oxidative stress and accelerates replicative and chronological aging of yeast cells devoid of anti-oxidative protection (with *SOD1* and *SOD2* gene deletion) and deprived of DNA repair mechanisms (*RAD52*). Interestingly, curcumin delays aging, probably through hormesis, of the wild-type strain BY4741.

## Introduction

Curcumin is a biologically active compound of vegetable origin. It was isolated for the first time from rhizomes of the perennial *Curcuma longa* plant (Vogel 1815). The compound has been widely used in food industry as a colourant or a spice (curcuma). However, curcumin raises interest primarily due to its predominantly pro-health action. The substance is characterised with a diverse biological activity related to anti-inflammatory, anti-oxidative, antimicrobial, pro-apoptotic and antineoplastic effect. Therefore, curcumin has had a very long tradition of use in natural medicine as it has no adverse effects. However, due to hydrophobic nature of the curcumin molecule, low absorption, and quick metabolism and elimination, the biological availability of curcumin is very low (Hewlings and Kalman 2017).

At the molecular level curcumin modulates a broad range of signalling molecules. It may increase or decrease their activity, depending on the target structure. The mechanism can be activated in two ways: by direct or indirect curcumin bounding. Indirect modulation refers to transcription factors, enzymes, inflammatory mediators, kinases, drug resistance proteins, adhesion molecules, growth factors, cell cycle regulation proteins and cell survival proteins. Direct action of curcumin refers to inflammatory molecules, kinases, reductases, histone acetyltransferases, integrins, DNA methyltransferase 1, carrier proteins and metal ions (Barchitta et al. 2019; Gupta et al. 2012).

The strong antioxidative effect of curcumin is connected with its ability to remove reactive oxygen species (ROS), including the dangerous hydroxyl radical, superoxide anion radical and nitric oxide (Toda et al. 1985). Moreover, it is able to activate genes of major antioxidant enzymes (Menon and Sudheer 2007). Furthermore, curcumin inhibits increase of the lipid peroxide level and protects lipids against oxidation (Wei et al. 2006).

Curcumin’s anti-inflammatory properties result from inhibiting activation of the inflammation factor NF-κB, which leads to lowering of inflammatory protein synthesis. Curcumin inhibits activation of the transcription factor through the IκB kinase complex (IKK), which is the NF-κB activator (Plummer et al. 1999). Antineoplastic properties of curcumin are connected with inhibition of cancer cell proliferation and induction of cell death (Duvoix et al. 2005). Curcumin stops the process of metastasis by inhibiting metalloproteinase activity (Aggarwal et al. 2005). Curcumin is also able to inhibit angiogenesis by lowering the expression of cytokines such as vascular endothelial growth factor and fibroblast growth factor (Arbiser et al. 1998). However, the most important anti-neoplastic property of curcumin is the ability to induce apoptosis and stop proliferation of cancer cells. With regard to neoplastic cells, the pro-apoptotic mechanism is related to induction of apoptosis through the mitochondrial pathway connected with oxidative stress, and through the intracellular pathway dependent on the p53 protein (Lantto et al. 2009; Shishodia and Aggarwal 2002).

Curcumin is also involved in regulation of the aging process. It may have an inhibiting effect on the TOR kinase and in this way delay aging (Beevers et al. 2006). Studies have shown a relationship between the TOR kinase and IKK involved in induction of inflammatory responses. As an IKK inhibitor, curcumin further blocks NF-κB as well as the TOR pathway, combining anti-inflammatory and anti-aging properties. Furthermore, the anti-oxidant action of curcumin related to improvement of the redox state in aging cells may have a positive impact on the delay of aging. Further details on curcumin impact on human organism can be found in the review paper (Hewlings and Kalman 2017).

Aging is a complex and multifactorial biological process that applies to all living organisms. Aging lowers an organism’s ability to respond to environmental stress. Over time, it triggers accumulation of intracellular damage and to impairment of tissue and organ function, eventually leading to the organism’s death. There have been numerous hypotheses and theories to explain the mechanisms of aging. The so called free radical theory of aging, which posits the destructive impact of reactive oxygen species (ROS) on the organism (Harman 1956), has been widely discussed for many years. ROS such as superoxide anion radicals lead to oxidation of cell macromolecules, which results in their malfunctioning. As such, the theory has as many advocates as it has opponents. Today we know that free radicals are not the main cause of aging and cell death; rather, they are one of many factors contributing to cell function distortion. Compounds that are capable of scavenging free radicals or transforming them into inactive forms are part of a defence mechanism preventing damage caused by free radicals.

Therefore, the purpose of this paper is to verify the anti-aging and anti-oxidative effects of curcumin using the *Saccharomyces cerevisiae* budding yeast as a model. We are the first to show impact of curcumin on the aging for yeast.

For the purpose of the study, we used the haploid yeast mutants *sod1Δ*, *sod2Δ* and *rad52Δ* in the BY4741 genetic background. CuZnSOD (*SOD1*) is the basic form of intracellular superoxide dismutase containing copper and zinc in the active site, occurring primarily in cytoplasm. *SOD1* enters the cell nucleus in response to oxidative stress to promote transcription of stress response genes. MnSOD (*SOD2*) located in the mitochondrial matrix contains manganese in the active site. The mitochondrial manganese superoxide dismutase protects cells against oxygen toxicity and oxidative stress (Fukai and Ushio-Fukai 2011). Rad52p participates in double-stranded DNA breaks repair. Although *RAD52* is expressed during the whole cell cycle, it is induced during meiosis as a response to DNA damage factors (Cole et al. 1989).

## Materials and methods

### Yeast strains

The strains used in this study are haploid wild-type yeast strain BY4741 (*MATa his3 leu2 met15 ura3*) and isogenic mutant strains *sod1Δ (MATa his3 leu2 met15 ura3 YJR104C::kanMX4)*, *sod2Δ* (*MATa his3 leu2 met15 ura3 YHR008C::kanMX4*), *rad52Δ* (*MATa his3 leu2 met15 ura3 YML032C::kanMX4*) (*EUROSCARF*).

### Growth conditions

Yeast cells were grown in a standard liquid YPD medium (1% Difco Yeast Extract, 1% Yeast Bacto-Peptone, 2% (w/v) glucose) without (control) or with 200 μM and 300 μM treatment curcumin on a rotary shaker at 150 rpm, or on a solid YPD medium containing 2% agar. The experiments were carried out at a the temperature of 28 °C.

### Kinetics of growth assay

Growth assays were carried out on liquid medium. Yeast cell suspension was incubated for 12 h in a shaking incubator at 28 °C (Heidolph Inkubator 1000 at 1200 rpm). The growth was monitored turbidimetrically in the Anthos 2010 type 17,550 microplate reader at 600 nm by performing measurements at 1 h intervals for 12 h. The data represent the mean values from three independent experiments.

### Determination of mean doubling time

The mean doubling time was calculated for each analyzed cell as described previously (Molon et al. 2016). The doubling time was calculated during the determination of the reproductive potential. The times of the first two reproductive cycles were not taken into account (the first and second doubling times are longer than those of older cells). The data represent the mean values from three independent experiments (with 45 cells used in each experiment) with a mean standard deviation (SD).

### Determination of budding lifespan

The budding lifespan of individual mother yeast cells was defined as the number of mitotic cycles (buddings) during the cell’s life. After overnight growth, cells were arrayed on a YPD plate without (control) or with 200 μM and 300 μM curcumin using a micromanipulator. The budding lifespan was determined microscopically by a routine procedure with the use of a micromanipulator as described previously (Molon et al. 2018). The number of buds formed by each mother cell signifies its reproductive potential (budding lifespan). In each experiment, forty-five single cells were analyzed. The results represent measurements for at least 90 cells analyzed in two independent experiments. The analysis was performed by micromanipulation using the Nikon Eclipse E200 optical microscope with an attached micromanipulator.

### Determination of total lifespan

The total lifespan was defined as the length of life of a single mother cell expressed in units of time. The total lifespan was calculated as the sum of reproductive and post-reproductive lifespans. The reproductive lifespan was defined as the length of time between the first and the last budding, and the post-reproductive lifespan as the length of time from the last budding until cell death. The lifespan of the Saccharomyces cerevisiae yeast was determined as previously described by (Minois et al. 2005) with small modification (Molon et al. 2018). Ten microliter aliquots of an overnight grown culture of yeast were collected and transferred on YPD plates with solid medium containing Phloxine B (10 μg/mL) and without (control) or with 200 μM and 300 μM curcumin. Phloxine B was used to stain *Saccharomyces cerevisiae* dead cells. Dead yeast cells lose membrane integrity and Phloxine B entered cell space giving pink/red coloration of cytosol. In each experiment, forty-five single cells were analysed. During manipulation, the plates were kept at 28 °C for 15 h and at 4 °C during the night. The results represent measurements for at least 90 cells analysed in two independent experiments. The analysis was performed by micromanipulation using the Nikon Eclipse E200 optical microscope with an attached micromanipulator.

### Chronological life span (CLS) assays

Chronological life span of cells incubated in minimal medium (SDC) was measured as described previously (Fabrizio and Longo 2003). Briefly, yeast were grown in SDC containing 2% (w/v) glucose, supplemented with histidine, leucine, methionine, uracil and appropriate concentration of curcumin. Chronological life span was monitored in expired SDC medium by measuring viability in 2, 5, 8, 12, 19 and 26 days. For the quantitative measurement of survival, staining with propidium iodide was used. The data represent the mean values from three independent experiments.

### Cell viability

For determining death cells staining with propidium iodide was used. Cells were suspended in PBS and stained with 5 μg/mL propidium iodide (Sigma-Aldrich) for 15 min in the dark at room temperature. Fluorescence pictures were taken with Olympus BX-51 microscope equipped with a DP-72 digital camera and cellSens Dimension software. Dead cells were red fluorescent. The data represent the mean values from three independent experiments.

### Estimation of cell diameter

Cell volume was estimated by optical microscopy and analysis of images collected in 2, 5, 8, 12 day during the routine procedure of determining the chronological lifespan. The images were captured with the Nikon Eclipse E200 microscope equipped with the Olympus DP26 digital camera. Cell diameter (d) live cells was measured using the Olympus cellSens Standard software in various planes for each cell and the mean value was used for calculations. The data represent the mean values from three independent experiments.

### Measurement of superoxide anion generation

Generation of reactive oxygen species (superoxide anion) was assessed with dihydroethidine (Invitrogen) (DHET; final concentration 18.9 μM) used standard protocols. The yeast cells from the exponential phase culture were washed with sterile water and suspended to the final density of 10^8^ cells/mL in 100 mM phosphate buffer pH 7.0 containing 0.1% (w/v) glucose and 1 mM sodium EDTA. The kinetics of fluorescence increase due to oxidation of the dihydroethidine was measured using the TECAN Infinite 200 microplate reader at λ_ex_ = 518 nm and λ_em_ = 605 nm at the temperature of 28 °C. The data represent the mean values from three independent experiments.

### Phenotypic analysis—a spot test for sensitivity to Congo red, Methyl methanesulfonate (MMS), sodium chloride (NaCl), Hydrogen peroxide (H_2_O_2_), Dodecyl sodium sulfate (SDS), Acetic acid and Heat shock

Yeast cultures were grown to exponential phase (OD_600nm_ between 0.8 and 1) and serially diluted to different cellular concentrations as indicated. Five microliters of each cell suspension was spotted onto agar plates containing curcumin or not (control) and various concentrations of Congo red (Sigma-Aldrich), methyl methanesulfonate (Sigma-Aldrich), sodium chloride (NaCl) (Sigma-Aldrich), Hydrogen peroxide (H_2_O_2_) (Sigma-Aldrich) (final concentration H_2_O_2_ 5 mM, incubation for 60 min with shaking), Dodecyl sodium sulfate (SDS) (Sigma-Aldrich), Acetic acid (Sigma-Aldrich) and Heat shock (incubation at 46 °C for 20 min). Growth was registered 48 h after incubation at 30 °C. All phenotypes described in this work were confirmed by three independent tests.

### Statistical analysis

The results represent the mean ± SD values for all cells tested in three or two independent experiments. The differences between the wild-type and the isogenic mutant strains were estimated using the one-way ANOVA and Dunnett’s post hoc test. The values were considered significant if *p < 0.05, **p < 0.01 or ***p < 0.001. Statistical analysis was performed using the Statistica 10 software.

## Results and discussion

### Impact of curcumin on the growth rate and mean doubling time

**Fig. 1:**
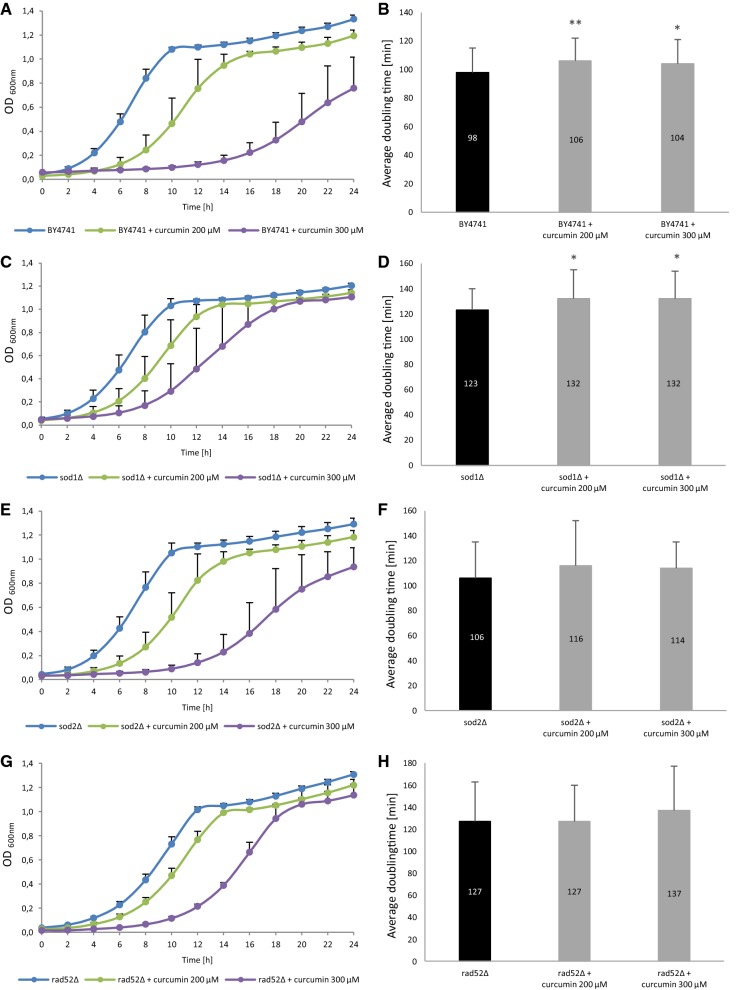
Comparison of growth kinetics (**a**, **c**, **e**, **g**) and average doubling time during reproducing (**b**, **d**, **f**, **h**) of the haploid wild-type yeast strain BY4741 and isogenic mutant strains *sod1Δ*, *sod2Δ*, *rad52Δ* treated with curcumin. The optical density (OD_600_) of the cultures was measured at different time points for up to 24 h. Error bars represent standard deviations obtained from three independent experiments Statistical significance (**b**, **d**, **f**, **h**) was assessed using ANOVA and the Dunnett’s post hoc test (*p < 0.05, **p < 0.01, ***p < 0.001) compared to the control (untreated). Bars indicate SD

First, we checked whether curcumin supplementation has impact on the growth rate of yeast cells. For that purpose, we obtained the 24-h growth curve for the analysed strains. For each strain, we determined the control curve with no curcumin added; then we measured the growth rate of cells treated with 200 µM and 300 µM curcumin, respectively. In all of the analysed strains curcumin supplementation resulted in clear inhibition of the growth rate (Fig. 1a, c, e, g). It should be emphasised that analysis of the growth curve shows the behaviour of the whole cell population, i.e. both the virgin cells with extended doubling time, which constitute ca. 50% of the population, and the older cells (Molon et al. 2016). This is why in addition to obtaining a standard 24-h growth curve for each tested strain with and without curcumin supplementation, we also measured the mean doubling time during the lifetime of cells (from birth until death). In the case of the wild-type BY4741 strain, supplementation of 200 µM and 300 µM curcumin resulted in a statistically significant extension of the mean doubling time, with p < 0.01 and p < 0.05, respectively (Fig. 1b). Similarly, analysis of the *sod1Δ* mutant showed that curcumin supplementation significantly extends the mean doubling time at p < 0.05 for both curcumin concentrations (Fig. 1d). In the case of the *sod2Δ* and *rad52Δ* mutants (Fig. 1f, h), we noted a small increase in the mean doubling time after curcumin supplementation; however, the increase was statistically insignificant compared to the control. A comparative analysis of these two methods of doubling time estimation shows clearly that the slowdown of growth, normally presented as a growth curve, does not always show real problems with growth of all yeast cells in a population. As shown previously, it is due to the slowdown of the first few buddings (Molon et al. 2016). Our data show that in the case of the analysed strains, in most of the cases the growth was inhibited after curcumin supplementation. These results are consistent with earlier reports. Studies with the use of yeast as a model organism unanimously show that *S. cerevisiae* is sensitive to this substance and that curcumin, through various pathways and mechanisms, inhibits its growth. Minear et al. have shown that curcumin inhibits growth of the *S. cerevisiae* yeast through iron chelation (Minear et al. 2011). Curcumin is an effective chelator of Fe(III). In their study, Minear et al. showed that curcumin inhibits growth of wild-type strains in a dose-dependent way, and that mutants with iron homeostasis defects are hypersensitive to curcumin. They conclude that curcumin penetrates yeast cells, concentrates in the endoplasmic reticulum membranes, and reduces the intracellular iron pool. Addition of iron to the medium improved substantially the growth of curcumin-sensitive strains (Minear et al. 2011). Also, addition of curcumin to the culture resulted in extending the doubling time and delaying the cell cycle progress depending on the supply of iron, as the concurrent supplementation of iron and curcumin alleviates negative effects of the delay in growth (Minear et al. 2011). Similar results related to the growth inhibition effect in yeast treated with curcumin and the reversal of that effect by addition of iron to the culture medium were obtained by Azad et al. (Azad et al. 2013). The latter also found out that curcumin acts as an intermediary in the activity of histone proteins and chromatin-modifying enzymes, as the mutants with histones devoid of N-terminal tails and with punctate mutations are hypersensitive to curcumin. Histone proteins and chromatin modifiers are the targets of curcumin activity and therefore curcumin may interfere with cell processes (Azad et al. 2013). Curcumin also acts in an epigenetic manner. The epigenetic regulatory role of curcumin is associated mainly with inhibition of DNA methyltransferases, regulation of histone modifications via regulation of histone acetyltransferases and deacetylases, and regulation of micro RNAs. Epigenetic regulation has been extensively reviewed elsewhere (Boyanapalli and Kong 2015). Further studies have shown that the yeast mitogen-activated protein kinase (MAPK) Hog1 is essential for the response to curcumin. In addition, curcumin, through the Hog1 protein, leads to up-regulation of the *GPD1* gene, which is necessary during osmotic stress response (Azad et al. 2014). Curcumin has been found to be biologically active against a variety of tumour types. It induces G2/M cell cycle arrest of head and neck squamous cell carcinoma and in colorectal cancer (Hu et al. 2017, Jaiswal et al. 2002). Curcumin has also been found to inhibit the growth of two pancreatic cancers cell lines in a dose- and time-dependent manner (Zhu and Bu 2017).

### Impact of curcumin on *Saccharomyces cerevisiae* yeast aging processes based on the replicative and chronological aging models

Most recent investigation provides vast amount of data on pro-health and anti-aging effects of curcumin on model organisms, yet so far little has been known on its impact on yeast aging. Therefore, we have been the first to measure the rate of aging of mitotically active yeast cells as well as yeast cells in the post-mitotic phase using the two accepted aging models, namely the replicative model and the chronological model.

First, we analysed curcumin impact on aging of mitotically active cells. In the standard replicative aging model, the length of life of the cell is defined as the number of daughters produced by the mother cell before the cell cycle stops irrevocably. Analyses based on that model are performed by means of physical separation of daughter cells from the mother cell under a microscope equipped with a micromanipulator (Kaeberlein 2010). In fact, the unit of replicative lifespan expresses the reproduction potential or fertility of a cell, not its real longevity. Therefore, in order to determine the actual length of life of a mitotically active cell, a new unit was proposed, namely the total lifespan (TLS), which is defined as the sum of the asexual reproduction lifespan and post-reproductive lifespan, or the time of life that passes from the last cell doubling until the cell’s death (Zadrag et al. 2008).

**Fig. 2:**
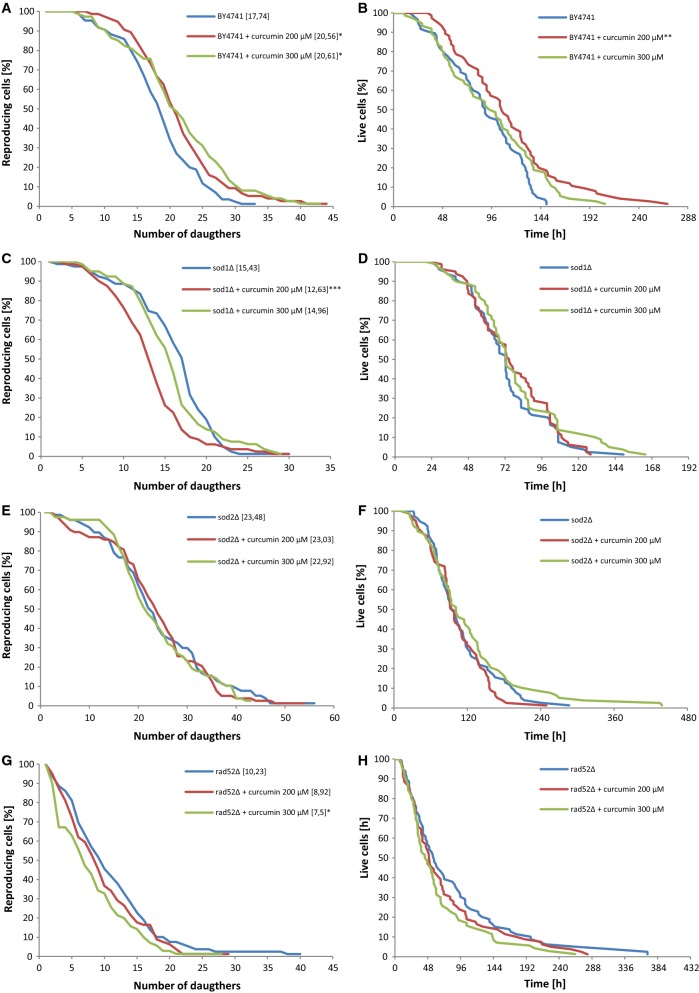
Comparison of the budding lifespan (**a**, **c**, **e**, **g**) and the total lifespan (**b**, **d**, **f**, **h**) of the haploid wild-type yeast strain BY4741 and isogenic mutant strains *sod1Δ*, *sod2Δ*, *rad52Δ* treated with curcumin. The values in parentheses are the mean values (for total 90 cells from two independent experiments) of the budding lifespan (**a**, **c**, **e**, **g**). Statistical significance was assessed using ANOVA and the Dunnett’s post hoc test (*p < 0.05, **p < 0.01, ***p < 0.001) compared to the control (untreated)

As seen in Fig. 2a, c, e, g, curcumin supplementation had an impact on reproductive potential only in the case of the wild-type strain (p < 0.05). In the case of the *sod1Δ* and *rad52Δ* mutants, curcumin statistically significantly decreased the reproductive potential of the cells (Fig. 2c, g), with p < 0.001 for *sod1Δ* at 200 µM curcumin concentration, and p < 0.05 for *rad52Δ* at 300 µM curcumin concentration. In the case of the *SOD2* gene deletion, curcumin supplementation reduced reproductive capacity of the cells; however, the changes were not statistically significant (Fig. 2e).

We have also measured the total lifetime of mitotically active cells, i.e. from the birth of the cell until its death. As seen in Fig. 2b, addition of 200 µM curcumin had a positive effect on the mean and maximum lifespans of the wild-type BY4741 yeast cells (p < 0.05). In the case of other strains (Fig. 2d, f, h), we noticed no significant impact of curcumin on the average lifespan. Furthermore, we determined the length of the period during which the cells were able to bud (reproductive lifespan) and the period during which they remained alive but unable to bud (post-reproductive lifespan). In the case of the wild-type BY4741 strain, supplementation of curcumin significantly extended the reproductive lifespan, with p < 0.01 for 200 µM curcumin and p < 0.05 for 300 µM curcumin, respectively (data not shown) A. In the case of the *rad52Δ* strain, addition of curcumin at the 300 µM concentration resulted in a significant reduction of the reproductive lifespan (p < 0.01) (data not shown).

Subsequently, we analysed the effect that curcumin had on chronological lifespan. Chronological lifespan is the measure of the length of survival time of the yeast population during the stationary phase or post-diauxic phase. Chronological lifespan is analysed with the use of a synthetic medium with supplementation of glucose and the necessary amino acids. When the exponential growth phase of the culture is completed, glucose concentration in the medium is exhausted and becomes very low, while yeast switches from fermentative metabolism to the aerobic respiration-based metabolism (diauxic shift). In addition, cells begin catabolic utilisation of the ethanol accumulated during fermentation and obtain energy from mitochondrial oxidative phosphorylation (Fabrizio and Longo 2003). Depletion of nutrients and the carbon source results in stopping of the cell cycle, which is necessary for the purpose of chronological lifespan analysis. Intracellular damage resulting from the production of ROS in mitochondria during respiration may accumulate over the course of chronological aging and prevent the cell from re-entering the cell cycle (Kaeberlein 2010).

**Fig. 3:**
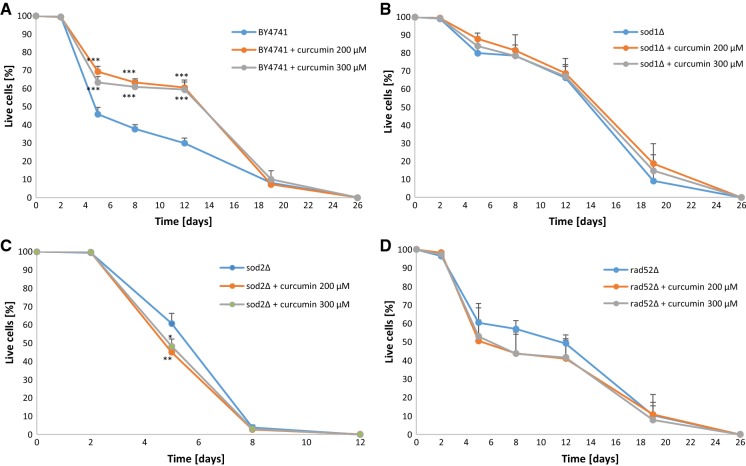
Chronological lifespan of the haploid wild-type yeast strain BY4741 (**a**) and isogenic mutant strains *sod1Δ* (**b**), *sod2Δ* (**c**), *rad52Δ* (**d**) treated with curcumin. Survival was determine by propidium iodide staining. Statistical significance was assessed using ANOVA and the Dunnett’s post hoc test (**p < 0.01, ***p < 0.001) compared to the control (untreated). Bars indicate SD

In the chronological aging model, curcumin supplementation had a significant positive impact on the survival rate of the wild-type BY4741 strain cells, with p < 0.001 at day 5, day 8 and day 12 of the experiment (Fig. 3a). In the case of the *sod2Δ* strain, addition of curcumin resulted in a significant decrease of the survival rate at day 5 of the experiment, with p < 0.05 after supplementation of 200 µM curcumin, and p < 0.01 after supplementation of 300 µM curcumin, respectively (Fig. 3c). For the *sod1Δ* and *rad52Δ* strains, the survival rate also dropped after curcumin supplementation, although not in a statistically significant manner (Fig. 3b, d).

**Fig. 4:**
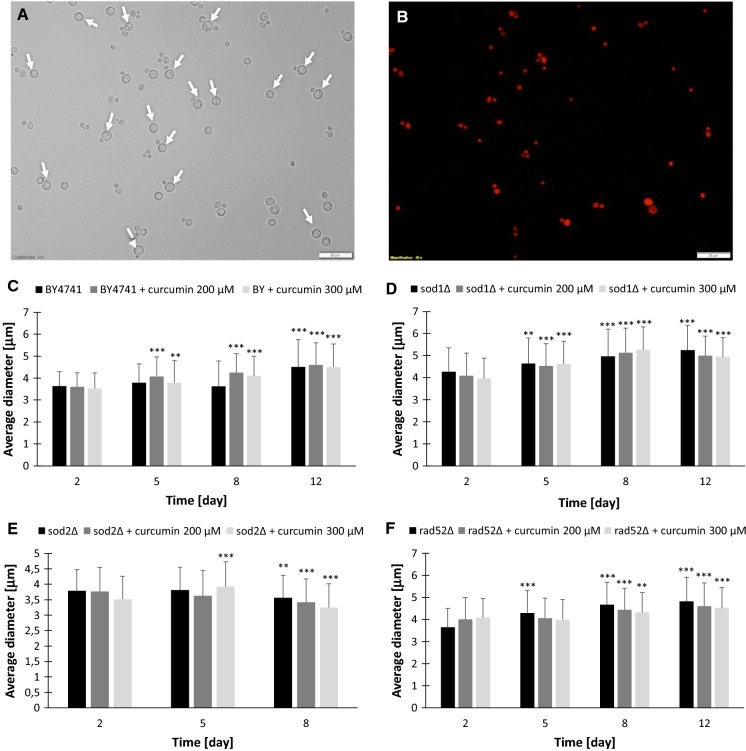
Changes in cells diameter during chronological lifespan. Only hypertrophic cells are able to survive. Representative image with propidium iodide staining of BY4741 with 200 mM curcumin treated (**a**, **b**). Cells growth up during stopping cell cycle (**c**–**f**). Statistical significance was assessed using ANOVA and the Dunnett’s post hoc test (**p < 0.01, ***p < 0.001) compared to the control (2 day). Bars indicate SD. Arrow indicate hypertrophic live cells

We also measured the cell size change kinetics over time. As shown in Fig. 4, in a post-mitotic culture the increase in cell size can be observed. We show that only hypertrophic cells are able to survive and are resistant to environmental factors, while small cells are sensitive to stress and die within a short period of time. We are the first to show that these results contradict the hypertrophy hypothesis, which postulates that cell hypertrophy has negative impact on cell function and survival of the organism (Bilinski and Bartosz 2006). Curcumin has also influence on human cells. It was shown that 50 μM curcumin induced apoptosis, caused cell cycle arrest in G1-phase and increased the volume of human colorectal adenocarcinoma HT-29 cells (Kossler et al. 2012).

It is worth emphasising that the curcumin concentrations used by us are much higher than those used in other studies, e.g. in mammalian cell lines. As shown by Minear et al. 2011, we can notice the growth inhibiting effect in yeast only from the concentration of approx. 150 μM.

Compared to yeast, the fruit fly (*Drosophila melanogaster*) has been much better and understood as a model organism for analysing impact of curcumin on aging and lifespan. Studies based on that model demonstrated that curcumin extended lifespan of the fruit fly (Lee et al. 2010). As shown by Lee et al., curcumin supplementation contributed to lifespan extension depending on gender and genotype (Lee et al. 2010). It did not affect nutrition habits or fertility of the tested subjects but had a protective effect against oxidative stress, with higher survival rates among individuals exposed to oxidants after prior curcumin treatment. Curcumin supplementation caused a delay in the onset of expression of age-related genes (Lee et al. 2010). Other studies also demonstrated that the average length of life of the fruit fly was extended in a dose-dependent way and that the effect was connected with the increased activity of superoxide dismutase (SOD), and therefore with protection against free radicals (Suckow and Suckow 2006). Also, Shen et al. noted fruit fly lifespan prolongation and increased SOD activity after curcumin supplementation. In addition, the level of malon dialdehyde (MDA) was evaluated. Average MDA levels in aging individuals (both male and female) were lowered in a way dependent on curcumin concentration and negatively correlated with age (Shen et al. 2013). Due to antioxidant properties of curcumin, its effect on viability of fruit flies exposed to radiation was also evaluated. It turned out that administration of curcumin to the tested individuals protects their cells against damage caused by radiotherapy and extends their lives, and therefore may provide effective protection against radiation (Seong et al. 2015). In addition to analysis of anti-oxidative action, the studies also included analysis of acetylcholinesterase (AChE) activity in relation to extension of the fruit fly’s life. A curcumin-enriched diet improved the survival capacity of the individuals; moreover, activity of superoxide dismutase and catalase increased while activity of acetylcholinesterase simultaneously decreased (Akinyemi et al. 2018).

The length of life of the *Caenorhabditis elegans* nematode may also be regulated by curcumin. Curcumin treatment may prolong the life of the adult individual and delay its aging (Liao et al. 2011). Particularly, the average and maximum lifespans are extended. The effect is ascribed to anti-oxidative properties of curcumin, as the intracellular level of ROS and lipofuscin was significantly reduced, which suggests that curcumin may slow down aging in the nematode. In addition, it was found out that curcumin has an impact on the size of the body and the esophageal pumping rate in nematodes. The length of the body in individuals receiving curcumin supplementation became shorter and the esophageal pumping rate became lower, which suggests that curcumin has an impact on nutritional behaviours of the nematode in the manner typical for calorie restriction. No changes in reproduction capacity were noted in comparison to the control. Furthermore, curcumin extended the lives of mutants hypersensitive to oxidative stress (Liao et al. 2011). Curcumin supplementation significantly increases survival of nematodes exposed to oxidative stress (Yu et al. 2014). It also prevents induced oxidative stress by intracellular scavenging of free radicals, including in mutants hypersensitive to oxidation. Exposure to curcumin strengthened expression of heat shock proteins (Yu et al. 2014). Extension of life and the related delay in aging in *C. elegans* due to curcumin is attributed mainly to its anti-oxidative properties and capability of reduction of the intracellular free radicals level.

Studies conducted on mammals confirm the argument of pro-health and anti-aging properties of curcumin. Bala et al. evaluated the impact on curcumin on parameters related to aging in brains of rats (Bala et al. 2006). Administration of turmeric to 6-month old and 24-month old rats resulted in a significant decrease in the content of lipid peroxides and lipofuscin in brain regions, while anti-oxidative enzymes such as superoxide dismutase (SOD), glutathione peroxidase and Na+/K+ -ATPase demonstrated a substantial increase in brain area activity. Changes in those age-related parameters show the anti-aging and neuroprotection properties of curcumin (Bala et al. 2006). The focus of the studies was also to determine whether curcumin can neutralise aluminium-induced aging changes in rats. The animals were administered curcumin and aluminium simultaneously; afterwards, their brains were measured to check for age-related parameters. It was found out that curcumin counteracted the neurotoxic action of aluminium (Sharma et al. 2009). Curcumin prevented aluminium-induced increase of lipid peroxidation and inhibition of activity of superoxide dismutase, glutathione peroxidase, glutathione transferase and Na+/K+ -ATPase in the brain areas of rats. Additionally, it prevented the increase in the activity of acetylcholinesterase and protein kinase *C* caused by aluminium (Sharma et al. 2009). Thus, curcumin has a protective effect against aluminium-induced strengthening of aging processes and age-related changes in rats by modulation of anti-oxidative parameters (Sharma et al. 2009).

Another target of curcumin which can contribute to alleviation of aging is the AMPK/UCP2 pathway (Pu et al. 2013). Ucp2 plays a key role in regulation of the production of reactive oxygen species and is connected with delay of aging process. Curcumin increases the AMPK and UCP2 phosphorylation levels in the brains of aging rats. Activation of the AMPK/UCP2 pathway by means of curcumin supplementation lowers the production of superoxide anion, while simultaneously increasing the production of nitric oxide in the endothelial cells of cerebral blood vessels. Because of its anti-oxidative action, curcumin reduces production of ROS and provides protection against dysfunction of cerebrovascular endothelium through the AMPK/UCP2 pathway in rodents and thus alleviates the progress of aging (Pu et al. 2013). The anti-aging role of curcumin in the mammalian model is also manifested by modulation of inflammation markers. Proinflammatory marker levels after curcumin treatment in rats were significantly lower compared to the control (Shailaja et al. 2017). In old rats treated with curcumin, anti-oxidative abilities and superoxide dismutase activity were higher, which shows that anti-oxidative activities of curcumin reduce inflammatory states related to age and that curcumin may have anti-aging effect (Shailaja et al. 2017). Curcumin delays aging and enhances fitness of aging rats mainly due to its anti-oxidative properties and improvement of the cell redox state.

### Impact of curcumin on the ROS level in yeast cells

**Fig. 5:**
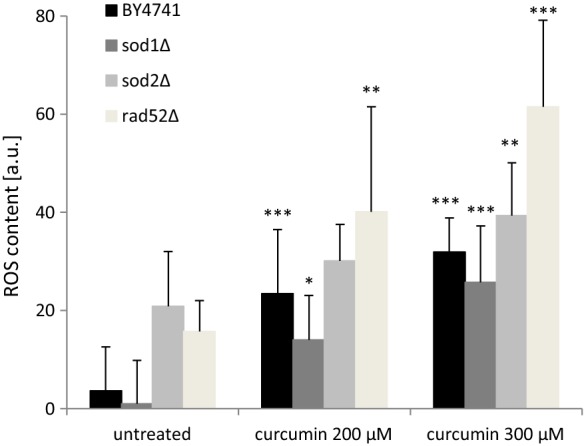
Superoxide anion content was estimated with the fluorescent probe dihydroethidine for yeast cells grown on liquid medium with curcumin supplementation. Statistical significance was assessed using ANOVA and the Dunnett’s post hoc test (**p < 0.01, ***p < 0.001) compared to the control (untreated). Bars indicate SD

Curcumin has often been described as an anti-oxidative substance. Our aim was to verify whether curcumin is capable of lowering the level of ROS in the *Saccharomyces cerevisiae* yeast cells. The results proved to be quite surprising. As seen in Fig. 5, curcumin supplementation at both concentrations caused a statistically significant increase in the level of ROS in the cell measured by DHE. Thus, we demonstrate that curcumin has doubtful anti-oxidative effect in the yeast model. We further support this statement by demonstrating the growth on the YPD medium with addition of curcumin after cell incubation with 5 mM H_2_O_2_, which is a strong oxidant. As shown in Table 1, the presence of curcumin also increases cell sensitivity to SDS, which, as demonstrated by Messina et al., also has oxidising properties (Messina et al. 2014). It seems that curcumin also increases cell resistance to Congo red in all of the analysed strains, and especially in the highest concentration, which indicates the influence of yeast cell wall biogenesis. Curcumin also decreases resistance of yeast cells, mainly the *sod1Δ* and *sod2Δ* mutants, to acetic acid. Interestingly, curcumin has little or no effect on osmotic and thermal stress. In general yeast has a rather low sensitivity because growth inhibition was achieved only with high concentrations, as shown in this study. Previous studies have shown that curcumin generates ROS in several cancer cells and triggers apoptosis (Patel et al. 2015, Zhang et al. 2015). Curcumin induced oxidative stress also in bovine leucocytes transformed by *Theileria annulata* (Araveti and Srivastava 2019). Our results may be explained by the fact that the effect of curcumin is strongly dependent on its concentration. Curcumin is a hormetin, which means that it can be beneficial at a low concentration but have a harmful effect at higher concentrations. Hormetins cause mild stress and eventually trigger hormesis, which is regarded as a promising strategy for slowing down the aging process and prevention or delay of age-related diseases. The presence of curcumin inside or outside the cell may be interpreted as a disorder or stress disrupting the cellular homeodynamics, thus initiating one or more stress response pathways in order to restore the homeodynamic balance (Demirovic and Rattan 2011). Numerous studies have shown that sensitivity to curcumin depends on the type of cell and, most probably, the cell cycle phase. In the in vitro studies, curcumin is toxic for all types of cells within a specific concentration range, while in another range it inhibits the cell cycle; at lower concentrations it seems to have no visible effect on cells (potentially beneficial doses according to curcumin activity).

**Table 1:** Sensitivity of the BY4741 strain and isogenic mutant strains *sod1Δ*, *sod2Δ*, *rad52Δ* treated with curcumin on cells stressed factors

Strain	Growth conditions								
	YPD	Congo red 100 μg/mL	MMS 0.03%	NaCl 1 M	H2O2 5 mM	SDS 0.01%	SDS 0.02%	Acetic acid 40 mM	Heat shock
BY4741	++++	++	+++	+++	+++	++++	+++	++++	++++
BY4741 + curcumin 200 µM	++++	++	+++	+++	++	++++	+++	++++	++++
BY4741 + curcumin 300 µM	++++	+++	+++	+++	++	+++	++	+++	++++
sod1Δ	++++	+	+++	+++	++	+++	+++	+++	++++
sod1 Δ + curcumin 200 µM	++++	+	+++	++	+	+++	++	++	++++
sod1 Δ + curcumin 300 µM	++++	++	+++	++	+	++	+	+	++++
sod2Δ	++++	++	+++	+++	++	+++	+++	+++	++++
sod2 Δ + curcumin 200 µM	++++	+++	+++	+++	+	+++	++	+++	++++
sod2 Δ + curcumin 300 µM	++++	+++	+++	+++	+	++	+	++	++++
rad52Δ	++++	+	−	+++	+	+++	+++	+++	++++
rad52 Δ + curcumin 200 µM	++++	++	−	+++	+	+++	++	+++	++++
rad52 Δ + curcumin 300 µM	++++	++	−	+++	+	++	+	+++	++++

Cells were cultured overnight in liquid YPD medium, counted and diluted serially to obtain suspensions at the densities 10^7^, 10^6^,10^5^ and 10^4^ cell/mL. Five microliters of each suspension was spotted at YPD solid medium containing various concentrations of curcumin and analyzed toxic factors. Plates were cultivated 2 days in 28 °C. Each “+” means the growth of one spot containing respectively: 50,000, 5000, 500, 50 cells

In conclusion, our studies for the first time indicate that curcumin can influence on yeast replicative and chronological aging. Curcumin accelerates replicative and chronological aging yeast cells devoid of anti-oxidative protection (with *SOD1* and *SOD2* gene deletion) and deprived of DNA repair mechanisms (*RAD52*) but not in the case wild-type cells. Our studies show that yeast cells can achieve longevity when antioxidant systems and DNA repair systems are working properly. We have further shown that hyperthrophic cells allow survive yeast during chronological aging probably via high resistance to enviromental stress factors, e.g. acetic acid. Additionally, curcumin induce oxidative stress in dose dependent manner. It seems that further research is necessary to prove that it is curcumin itself and not the breakdown products of this compound that have a beneficial effect on cells.
